# Presumed Relapse of Plasmodium vivax Malaria Presenting With Thrombocytopenia in a Non-endemic Area: A Case Report

**DOI:** 10.7759/cureus.111347

**Published:** 2026-06-23

**Authors:** Kezia Febiola P Dewi, Dwi P Mulyani

**Affiliations:** 1 Department of Emergency Medicine, Sentosa Mother and Child Hospital, Makassar, IDN; 2 Department of Emergency Medicine, Sayang Ibu Mother and Child Hospital, Balikpapan, IDN

**Keywords:** case report, febrile illness, malaria relapse, non-endemic area, plasmodium vivax, thrombocytopenia, tropical disease

## Abstract

Malaria remains a significant global health problem, particularly in tropical regions. Although *Plasmodium vivax* infection is often considered benign, it possesses the unique ability to cause relapse through dormant liver stages known as hypnozoites. Delayed diagnosis may occur in non-endemic or low-incidence settings because malaria can mimic more common febrile illnesses such as dengue infection.

We report a case of presumed relapse of *P. vivax* malaria in a 54-year-old female presenting in a non-endemic area with cyclical fever, chills, sweating, headache, nausea, and vomiting. The patient had a prior history of malaria infection one year earlier. Initial laboratory evaluation revealed significant thrombocytopenia with a platelet count of 47000/µL, raising initial suspicion of dengue fever. However, dengue NS1 antigen testing was negative. Species-specific malaria rapid diagnostic testing *Plasmodium falciparum*/*P. vivax* antigen (Pf/Pv Ag) and peripheral blood smear examination using Giemsa staining subsequently confirmed *P. vivax* infection.

The patient was treated with dihydroartemisinin-piperaquine (DHP) followed by primaquine for radical cure, resulting in clinical and hematologic improvement.

This case highlights the diagnostic challenges of presumed malaria relapse in non-endemic settings, particularly when overlapping clinical and hematological features with dengue infection may lead to initial misdiagnosis. Thorough travel history assessment, early recognition, and appropriate diagnostic testing are essential to ensure timely treatment and prevent complications.

## Introduction

In Indonesia, malaria remains a public health concern, particularly in eastern provinces such as Southeast Sulawesi. However, the malaria incidence rate in Konawe Regency, Southeast Sulawesi, was reported to be relatively low at 0.02 cases per 1000 population in 2023, which may contribute to lower clinical suspicion and potential diagnostic delay in low-incidence settings [1]. Malaria is caused by protozoan parasites of the genus Plasmodium and transmitted through the bite of infected female Anopheles mosquitoes. Among the species infecting humans, *Plasmodium vivax* is the most widely distributed and is traditionally associated with a milder clinical course compared to *Plasmodium falciparum* [2].

*P. vivax* remains a major contributor to the global malaria burden, particularly in Asia and other tropical regions. Although often considered less severe than *P. falciparum*, delayed diagnosis and treatment may still result in significant morbidity, especially in low-incidence or non-endemic settings where clinical suspicion is reduced. In malaria, relapse refers to reactivation of dormant liver hypnozoites, whereas recrudescence results from incomplete clearance of blood-stage parasites, and reinfection occurs following a new mosquito inoculation [3].

However, *P. vivax* possesses a unique biological characteristic that distinguishes it from other species: the ability to form dormant liver stages known as hypnozoites. These latent forms can reactivate weeks to months after the initial infection and may result in relapse even in the absence of a new mosquito bite [4]. This feature presents a diagnostic challenge, particularly in non-endemic or low-incidence areas where malaria may not be initially suspected because it is encountered less frequently, resulting in lower clinical suspicion among clinicians. In addition, the nonspecific presentation of malaria may overlap with common febrile illnesses such as dengue, including manifestations such as fever, headache, nausea, vomiting, and thrombocytopenia. Previous studies have reported thrombocytopenia in more than 60% of malaria cases and highlighted the overlapping clinical and hematological features between malaria and dengue, which may contribute to delayed diagnosis and mismanagement [5,6].

This report aims to present a case of presumed relapse of *P. vivax* malaria presenting with thrombocytopenia in a low-incidence area, highlighting the importance of early recognition and accurate diagnosis.

## Case presentation

In June 2025, a 54-year-old female presented to the emergency department with a three-day history of cyclical fever accompanied by chills, sweating, headache, nausea, and vomiting. There were no complaints of diarrhea or urinary symptoms. The patient had only taken paracetamol with no clinical improvement.

Further history-taking revealed that the patient had stayed in Papua, an endemic malaria region, for one month approximately one year prior to presentation, without receiving malaria prophylaxis. During that period, she experienced a febrile illness and was treated with antimalarial therapy. The patient had no history of blood transfusion.

On physical examination, the patient appeared moderately ill and was fully conscious (compos mentis). Vital signs revealed a temperature of 38.3°C, blood pressure of 119/69 mmHg, pulse rate of 103 beats/minute, respiratory rate of 22 breaths/minute, and oxygen saturation of 98% on room air. There were no accompanying respiratory symptoms such as cough or dyspnea. Hydration and peripheral perfusion were considered adequate, with capillary refill time <2 seconds and no peripheral edema. Abdominal examination demonstrated mild epigastric tenderness without hepatosplenomegaly.

Initial hematologic evaluation revealed thrombocytopenia (platelet count: 47×10³/µL), mild anemia with hemoglobin of 11 g/dL, hematocrit of 31.2%, and a leukocyte count of 8.1×10³/µL. Given the local epidemiology, dengue fever was initially considered the primary differential diagnosis. Dengue NS1 antigen testing was negative. Species-specific malaria rapid diagnostic testing *P. falciparum*/*P. vivax* antigen (Pf/Pv Ag) was positive for *P. vivax*.

**Figure 1:**
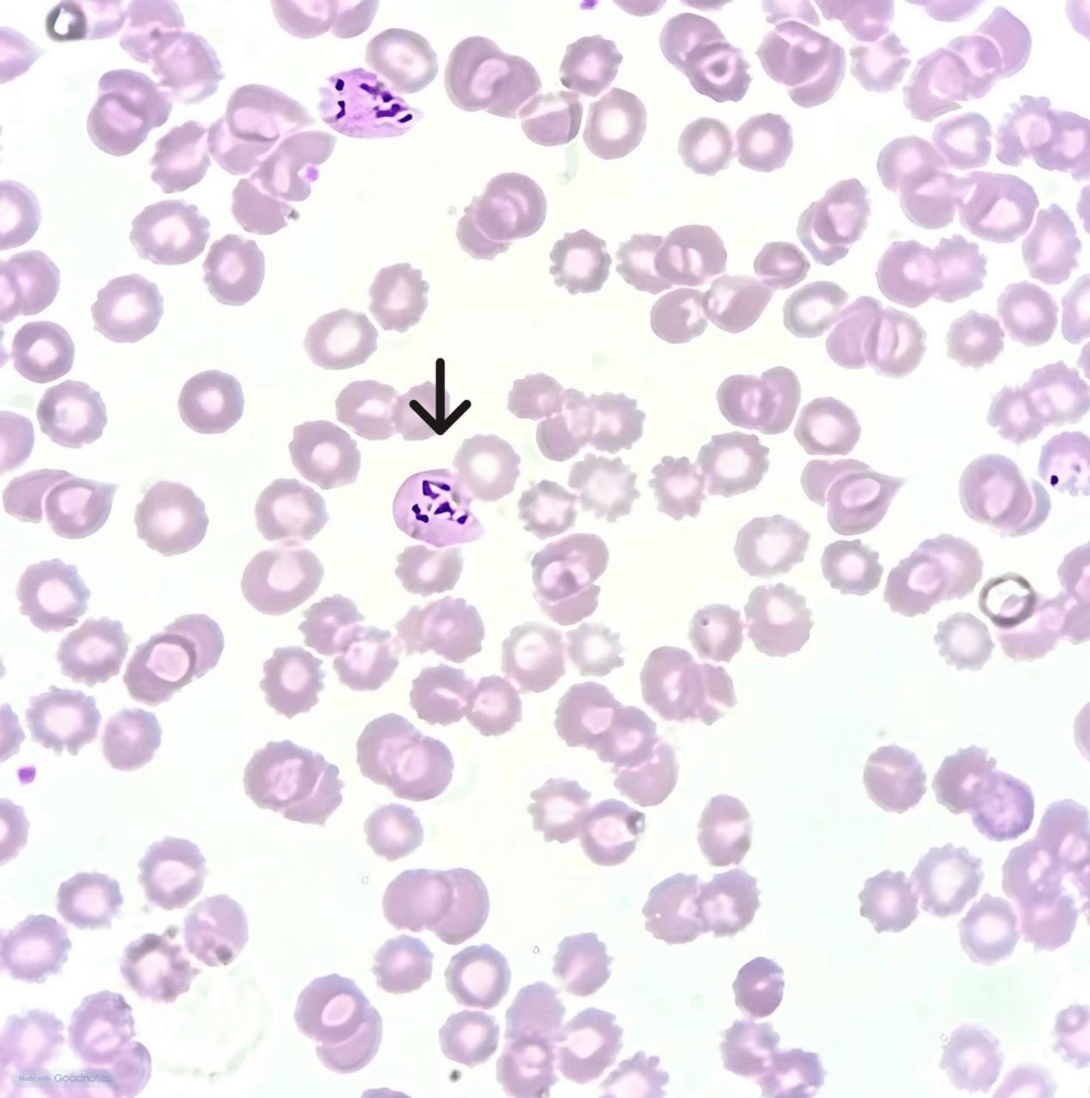
Giemsa-stained peripheral blood smear demonstrating intraerythrocytic ring forms and trophozoites of Plasmodium vivax (black arrow) (1000× magnification)

**Figure 2:**
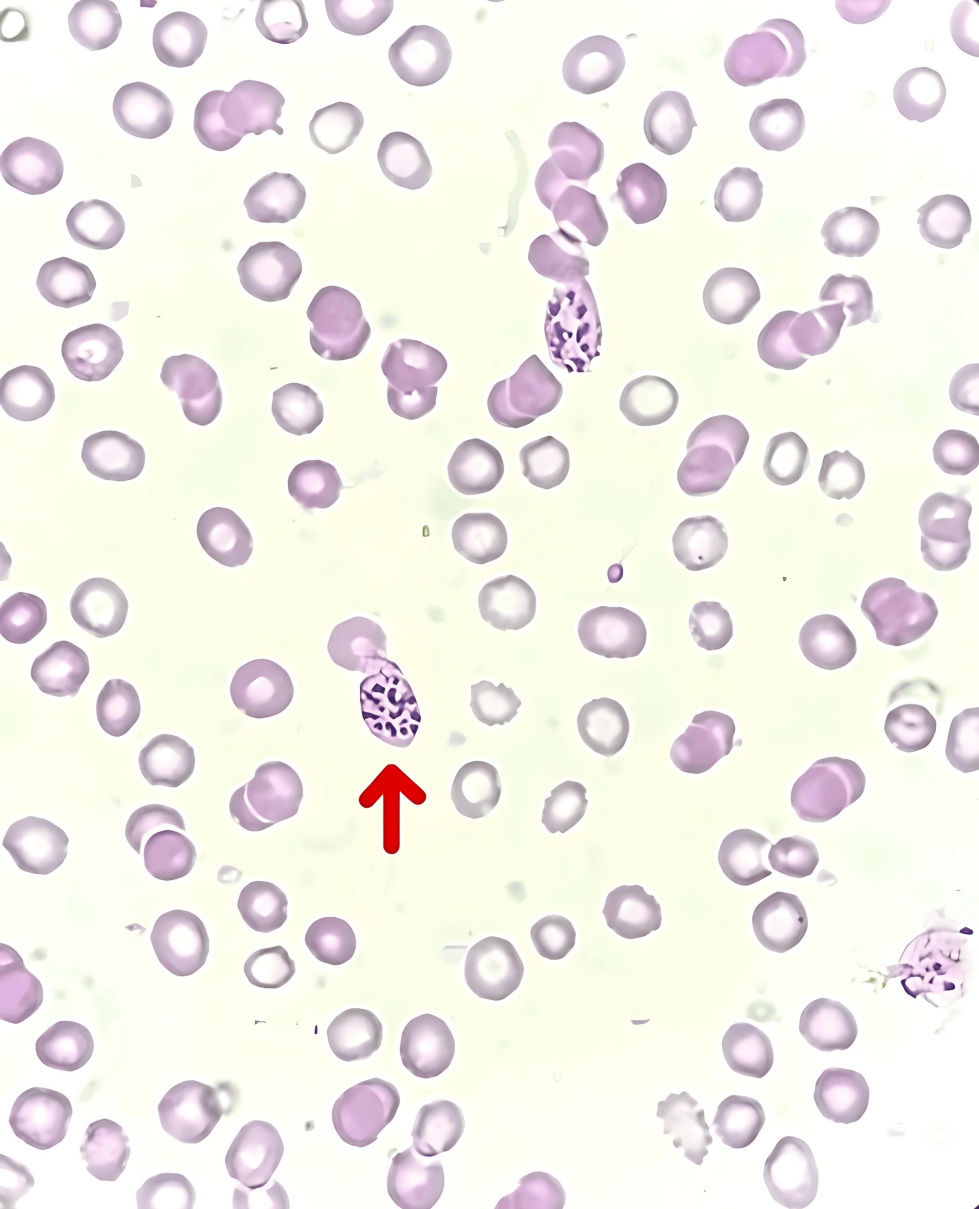
Giemsa-stained peripheral blood smear demonstrating schizont forms of Plasmodium vivax with multiple merozoites within infected erythrocytes (red arrow) (1000× magnification)

Peripheral blood smear examination using Giemsa staining confirmed *P. vivax* infection, demonstrating intraerythrocytic ring forms and trophozoites (Figure 1), as well as schizont forms with multiple merozoites within infected erythrocytes (Figure 2).

A diagnosis of presumed relapse of *P. vivax* malaria was established. The patient received intravenous crystalloid fluids for hydration, intravenous paracetamol 1 g every eight hours as needed for fever, intravenous omeprazole 40 mg every 12 hours, and intravenous ondansetron 4 mg every eight hours for symptomatic management. Antimalarial therapy consisted of oral dihydroartemisinin-piperaquine (DHP) (40 mg/320 mg per tablet), three tablets once daily for three consecutive days, followed by oral primaquine 15 mg once daily for 14 days as a radical cure.

During hospitalization, the patient was monitored clinically with serial assessment of vital signs and hematologic parameters. After three days of treatment, the platelet count improved from 47000/µL to 78000/µL. The patient demonstrated gradual clinical improvement with complete resolution of fever, chills, headache, nausea, and vomiting. She remained hemodynamically stable throughout hospitalization, with no evidence of bleeding or other complications. On hospital day 4, she was discharged in stable condition with normal vital signs, good oral intake, and instructions to complete the remaining course of primaquine.

At the first outpatient follow-up one week after discharge, the patient remained clinically well, with no recurrence of fever, bleeding manifestations, or other symptoms, and reported good adherence to the prescribed primaquine regimen without adverse effects. At the second follow-up two weeks after discharge, she had completed the 14-day course of primaquine without adverse effects. She remained asymptomatic, and repeat laboratory evaluation demonstrated normalization of the platelet count.

## Discussion

*P. vivax* malaria has traditionally been regarded as a benign infection. However, accumulating evidence has demonstrated that it may cause severe thrombocytopenia, organ dysfunction, and recurrent episodes even in non-endemic settings. Our patient similarly presented with marked thrombocytopenia despite the absence of severe bleeding or organ failure [7,8].

Malaria in low-endemic or non-endemic areas may be overlooked because of its nonspecific presentation, leading to delayed diagnosis and management. Similar diagnostic challenges have been described in previous reports of imported or relapsing *P. vivax* malaria, in which patients initially underwent evaluation for dengue because both diseases commonly present with acute febrile illness and thrombocytopenia. In our patient, thrombocytopenia initially raised suspicion of dengue infection because dengue is more prevalent in our setting. However, the cyclical fever pattern, history of travel to Papua, negative dengue NS1 antigen test, and positive peripheral blood smear supported the diagnosis of malaria. This emphasizes the importance of obtaining a detailed travel history when evaluating patients with undifferentiated febrile illness in low-endemic settings [8,9].

The present case was considered most consistent with presumed relapse of *P. vivax* malaria because the patient had documented previous malaria infection, had not revisited an endemic area, and presented approximately one year after the initial episode. Similar delayed relapses have been described in travelers returning from endemic regions, although the interval between the primary infection and relapse varies considerably depending on parasite strain, geographic origin, host immunity, and adequacy of previous radical cure. Because molecular genotyping was unavailable, relapse could not be distinguished definitively from recrudescence or reinfection; therefore, the diagnosis remained presumptive [9].

Severe thrombocytopenia was the most prominent laboratory abnormality in our patient (47×10⁹/L), yet she did not develop clinically significant bleeding. This finding is consistent with previous studies and case reports demonstrating that thrombocytopenia is common in *P. vivax* malaria but does not necessarily predict hemorrhagic complications. Proposed mechanisms include immune-mediated platelet destruction, splenic sequestration, oxidative stress, and reduced platelet survival. Therefore, thrombocytopenia should be interpreted together with clinical findings and confirmatory malaria testing rather than as an isolated indicator of disease severity [7,9]. Similar to the Romanian case report [9], our patient developed severe thrombocytopenia without clinically significant bleeding. In contrast, the Brazilian pediatric case demonstrated only mild thrombocytopenia despite a comparable relapse episode [10]. This variability suggests that platelet decline may be influenced by host immune response and parasite burden rather than relapse itself.

The patient responded favorably to treatment with DHP followed by primaquine, with rapid resolution of fever and normalization of platelet counts at two-week follow-up. This favorable clinical course is comparable to outcomes reported in previous cases when radical cure was completed. Current guidelines recommend artemisinin-based combination therapy (ACT) for blood-stage infection together with primaquine for eradication of dormant hypnozoites, provided that glucose-6-phosphate dehydrogenase (G6PD) deficiency has been excluded whenever possible [11,12].

As summarized in Table 1, our patient shared several clinical features with previously published cases of presumed relapse of *P. vivax* malaria, including recurrent fever, thrombocytopenia, positive peripheral blood smear findings, and a favorable clinical response following radical cure. However, our patient experienced a longer interval between the initial infection and presumed relapse (approximately one year) than the previously reported cases (6-8 months). This difference may reflect variations in parasite strain, host immune response, adequacy of prior radical cure, or geographic differences in hypnozoite latency. Furthermore, despite severe thrombocytopenia, no clinically significant bleeding occurred, consistent with previous reports demonstrating that thrombocytopenia does not necessarily predict hemorrhagic complications [9,10,12].

**Table 1:** Comparison of the present case with previously published case reports of relapsing Plasmodium vivax malaria in non-endemic settings DHP, dihydroartemisinin-piperaquine; ACT, artemisinin-based combination therapy

Characteristic	Romania (2025)	Brazil (2024)	Present case
Age/sex	46/M	12/F	54/F
Clinical setting	Romania	Brazil	Indonesia (low-endemic area)
Previous malaria history	Yes	Yes	Yes
Interval to relapse	8 months	6 months	~1 year
Fever	Yes	Yes	Yes
Thrombocytopenia	Severe	Mild	Severe (47×10⁹/L)
Bleeding	No	No	No
Peripheral blood smear	Positive	Positive	Positive
Treatment	ACT+primaquine	Chloroquine+primaquine	DHP+primaquine
Outcome	Recovered	Recovered	Recovered

This case has several limitations. The diagnosis of presumed relapse was based on the patient’s prior history of malaria infection and the absence of recent travel to endemic areas; however, definitive differentiation between relapse, recrudescence, and reinfection could not be established because molecular testing and prior species documentation were unavailable. In addition, quantitative parasitemia assessment, comprehensive biochemical testing, and G6PD deficiency screening prior to primaquine administration were not available due to limited resources in our remote district hospital setting, which serves as the only district referral hospital in the region. Nevertheless, negative dengue NS1 antigen testing and confirmatory peripheral blood smear findings supported the diagnosis of *P. vivax* malaria. This case highlights the importance of maintaining a high index of suspicion for presumed malaria relapse in patients presenting with febrile illness outside endemic regions, particularly in those with a history of prior infection or travel to endemic areas.

## Conclusions

This case illustrates that presumed relapse of *P. vivax* malaria may mimic dengue because of overlapping clinical manifestations and thrombocytopenia. Careful assessment of travel history and prompt malaria testing are essential for early diagnosis and appropriate treatment, even in low-endemic settings.

## References

[1] Naser RH, Rajaii T, Farash BR, javad Seyyedtabaei S, Hajali V, Sadabadi F, Saburi E (2026). Disease cases by regency/city and type of disease, 2023 (Indonesian). Mol Biochem Parasitol.

[2] Menkin SL, Koval K, Samandari T (2026). Plasmodium vivax Malaria. Int J Sci Res Innov Stud.

[3] Singh D, Uddin A, Bajaj K (2025). Clinical spectrum and complications of Plasmodium vivax malaria: a retrospective study from Delhi, India. Malariaworld J.

[4] Sahu PK, Sahu PS, Ahmed A, Ambu S (2016). A review of concurrent infections of malaria and dengue in Asia. Asian Pac J Trop Biomed.

[5] Castillo-Fernández N, Soriano-Pérez MJ, Lozano-Serrano AB (2025). Pre-hospital time delays in imported malaria diagnosis in hospitalized sub-Saharan travelers and migrants: not only on the patient’s shoulders. Infection.

[6] Senduk EB, Tuda JSB, Kurniawan A, Diptyanusa A (2025). Correlation of hematological profiles and parasite density of malaria patients from low endemic areas in North Sulawesi, Indonesia. eJ Kedokt Indones.

[7] Achame MS, Gedefie A, Debash H, Tesfaye A, Tiruneh KT, Kassaw AB (2025). Prevalence of thrombocytopenia among patients with malaria in Ethiopia: a systematic review and metanalysis. Malar J.

[8] Siddig EE, Mohamed NS, Ahmed A (2024). Severe coinfection of dengue and malaria: a case report. Clin Case Rep.

[9] Andrejkovits ÁV, Pop AV, Fejér M, Gîrbovan EC, Coșeriu RL, Vintilă C, Văsieșiu AM (2025). Recurrent malaria with Plasmodium vivax: a case report and brief review of the literature. Trop Med Infect Dis.

[10] Martins EB, de Pina-Costa A, Mamani RF (2024). Relapsing Plasmodium vivax malaria in a 12-year-old Brazilian girl: a case report. Malariaworld J.

[11] Antinori S, Giacomelli A, Casalini G, Ridolfo AL (2024). How to manage adult patients with malaria in the non-endemic setting. Clin Microbiol Infect.

[12] Chu CS, White NJ (2016). Management of relapsing Plasmodium vivax malaria. Expert Rev Anti Infect Ther.

